# Inhibition and Facilitation of the Spinal Locomotor Central Pattern Generator and Reflex Circuits by Somatosensory Feedback From the Lumbar and Perineal Regions After Spinal Cord Injury

**DOI:** 10.3389/fnins.2021.720542

**Published:** 2021-07-28

**Authors:** Angèle N. Merlet, Jonathan Harnie, Alain Frigon

**Affiliations:** Department of Pharmacology-Physiology, Faculty of Medicine and Health Sciences, Centre de Recherche du CHUS, Université de Sherbrooke, Sherbrooke, QC, Canada

**Keywords:** locomotion, somatosensory feedback, perineal, lumbar, spinal cord injury, spinal reflexes, cutaneous, micturition

## Abstract

Somatosensory feedback from peripheral receptors dynamically interacts with networks located in the spinal cord and brain to control mammalian locomotion. Although somatosensory feedback from the limbs plays a major role in regulating locomotor output, those from other regions, such as lumbar and perineal areas also shape locomotor activity. In mammals with a complete spinal cord injury, inputs from the lumbar region powerfully inhibit hindlimb locomotion, while those from the perineal region facilitate it. Our recent work in cats with a complete spinal cord injury shows that they also have opposite effects on cutaneous reflexes from the foot. Lumbar inputs increase the gain of reflexes while those from the perineal region decrease it. The purpose of this review is to discuss how somatosensory feedback from the lumbar and perineal regions modulate the spinal locomotor central pattern generator and reflex circuits after spinal cord injury and the possible mechanisms involved. We also discuss how spinal cord injury can lead to a loss of functional specificity through the abnormal activation of functions by somatosensory feedback, such as the concurrent activation of locomotion and micturition. Lastly, we discuss the potential functions of somatosensory feedback from the lumbar and perineal regions and their potential for promoting motor recovery after spinal cord injury.

## Introduction

Terrestrial locomotion in mammals is a complex movement that engages all levels of the nervous system. The basic pattern of locomotion, consisting of flexor and extensor alternation in one limb and left-right alternation, is generated at the level of the spinal cord by a network of neurons, the so-called central pattern generator (CPG) (reviewed in Grillner, 1981; Rossignol et al., 2006; McCrea and Rybak, 2008; Kiehn, 2016; Frigon, 2017; Grillner and El Manira, 2020). The spinal locomotor CPG receives inputs from peripheral mechanoreceptors located in muscles, tendons, joints and skin, collectively termed somatosensory feedback, as well as from various supraspinal structures. Somatosensory feedback provides information related to the internal state of the body (proprioceptive) and external physical conditions (tactile). Supraspinal structures also receive somatosensory feedback and inputs related to vision, body orientation (vestibular) and cognitive processes. Locomotion is generated through the complex interplay between these control systems.

Complete spinal cord injury (SCI), such as a spinal transection, permanently abolishes all communication between the brain and spinal networks controlling leg movements. However, because of the spinal locomotor CPG and its interactions with somatosensory inputs, various sensorimotor functions recover, such as spinal reflexes and locomotion, in various mammals, such as mice, rats and cats, and even humans but to a lesser degree (Rossignol and Frigon, 2011). Even more remarkable, cats with a spinal transection (i.e., spinal cats) can adjust to treadmill speed (Hurteau et al., 2017; Harnie et al., 2018, 2019), left-right speed differences on a split-belt treadmill (Frigon et al., 2017; Kuczynski et al., 2017), stepping on an incline (Higgin et al., 2020) and stepping backward when the treadmill is reversed (Harnie et al., 2021).

Although somatosensory feedback from the limbs shapes the output of the spinal locomotor CPG, inputs from other body regions also generate powerful inhibition or facilitation of locomotion. For instance, inputs from the lumbar skin inhibit hindlimb locomotion (Viala and Buser, 1974; Viala et al., 1978; Frigon et al., 2012b; Hurteau et al., 2015; Merlet et al., 2020), while inputs from the skin of the perineal region facilitate it (Barbeau and Rossignol, 1987; Belanger et al., 1996; Leblond et al., 2003; Langlet et al., 2005; Hochman, 2012; Alluin et al., 2015; Harnie et al., 2019). Our recent work in chronic spinal cats shows that somatosensory inputs from the lumbar and perineal regions, respectively, increase and decrease the gain of cutaneous reflexes from the foot (Merlet et al., 2020, 2021). Thus, lumbar and perineal inputs have opposite effects on reflexes, weight support and locomotor activity. The purpose of this review is to discuss how somatosensory feedback from the lumbar and perineal regions modulate the spinal locomotor central pattern generator and reflex circuits, the possible mechanisms involved and their potential functions. We also discuss the loss of functional specificity in response to somatosensory feedback after SCI. We finish by discussing the functional roles of somatosensory feedback from lumbar and perineal regions and its potential to promote the recovery of motor functions after SCI.

## Somatosensory Feedback Inhibits or Facilitates Hindlimb Locomotion

During locomotion, somatosensory feedback from receptors located in muscles, joints, and skin of the limbs interacts dynamically with the spinal locomotor CPG to regulate phase durations and transitions as well as to change the magnitude of muscle activity to meet task demands (reviewed in Duysens et al., 2000; Dietz, 2002; Rossignol et al., 2006; Pearson, 2008). For example, electrically stimulating the ankle extensor nerve at group I strength resets the rhythm from flexion to extension or prolongs the ongoing extensor burst during fictive locomotion in decerebrate cats (Conway et al., 1987; Gossard et al., 1994; Guertin et al., 1995; Schomburg et al., 1998; Frigon et al., 2010). Simulating flexor muscle afferents and cutaneous afferents from the foot also resets or entrains the fictive locomotor rhythm (Duysens, 1977; Guertin et al., 1995; Perreault et al., 1995; Schomburg et al., 1998; Lam and Pearson, 2001; Stecina et al., 2005; Frigon et al., 2010). Resetting and entraining the locomotor rhythm suggest that inputs have direct access to spinal rhythm-generating circuitry (Conway et al., 1987; Hultborn et al., 1998; Schomburg et al., 1998; McCrea and Rybak, 2008; Pearson, 2008; Gossard et al., 2011; Frigon et al., 2012b). In humans, somatosensory feedback from the limbs also interacts with spinal networks (reviewed in Duysens et al., 2000; Dietz, 2002). For example, afferents that signal hip-joint position are critical for H-reflex modulation at various hip angles (Chapman et al., 1991; Brooke et al., 1993; Knikou and Rymer, 2002b), and for switching reflex actions from inhibitory to facilitatory (Knikou and Rymer, 2002a). Although somatosensory inputs from the limbs plays a major role in regulating the locomotor rhythm, those from the lumbar and perineal regions also interact with the spinal locomotor CPG to shape locomotor activity.

### Inhibition of the Spinal Locomotor CPG With Mechanical Stimulation of the Lumbar Region

Studies in rabbits and cats have shown that activating mechanoreceptors of the lumbar region inhibits spinal neuronal circuits that generate weight support and locomotion (Viala and Buser, 1974; Viala et al., 1978; Frigon et al., 2012b; Hurteau et al., 2015; Merlet et al., 2020). In curarized decerebrate rabbits with intact or transected spinal cords, mechanical or selective electrical stimulation of a dorsal lumbosacral cutaneous nerve, at an intensity that recruits Aδ fibers, instantly abolished fictive locomotor-like activity (Viala and Buser, 1974; Viala et al., 1978). More recently, it was shown that pinching the lumbar skin abolished locomotor-like activity during terminal experiments in chronic spinal decerebrate cats treated with clonidine, an α2-noradrenergic agonist known to facilitate hindlimb locomotion or locomotor-like activity in spinal cats (Frigon et al., 2012b). In this study, however, the hindlimbs were restrained. Pinching the lumbar skin resets the locomotor-like rhythm to flexion and maintained it in flexion. In another study in awake chronic spinal cats not treated pharmacologically, also with the hindlimbs restrained, mechanically stimulating the lumbar region with vibration or manual pinch abolished locomotor-like activity initiated by electrically stimulating the superficial peroneal or distal tibial nerve (Merlet et al., 2020). During treadmill locomotion, pinching the lumbar skin abolished weight support and hindlimb movements in chronic spinal cats (Hurteau et al., 2015). In rabbits and cats, the powerful inhibitory effect persists as long as the mechanical stimulation is applied. In humans, the inhibitory mechanism from the dorsal lumbar region to spinal locomotor networks appears to have been conserved because pinching the lumbar skin in a subject with a motor complete SCI abolished spontaneous involuntary rhythmic activity (Nadeau et al., 2010).

The most consistent inhibitory effects were observed with cutaneous stimulation centered at mid lumbar levels, from L2 to L5 in rabbits and cats (Viala and Buser, 1974; Hurteau et al., 2015). The area of effective stimulus is large in rabbits (> 0.3 cm^2^) (Viala and Buser, 1974) and cats (> 1 cm^2^) (Hurteau et al., 2015). In rabbits, the inhibitory effect was attributed to the activation of Aδ fibers from the lumbosacral skin (Viala et al., 1978). This study performed two experiments where they 1) recruited different groups of fibers through a progressive increase in electrical stimulation intensity and 2) isolated selective groups of fibers (Viala et al., 1978). In both experiments, the activation of Aδ fibers was clearly inhibitory whereas the activation of other afferents was moderately (Aα, Aβ) or strongly (C fibers) excitatory (Viala et al., 1978). However, a slight pressure to the lumbar region was sufficient to reduce weight support in chronic spinal cats (Hurteau et al., 2015), indicating the involvement of non-nociceptive afferents from low-threshold mechanoreceptors in the inhibition. Thus, it is possible that Aα and Aβ fibers are also involved. Moreover, although the majority of Aδ fibers are high threshold nociceptive afferents, a subset of these fibers responds to non-nociceptive low threshold stimuli (Abraira and Ginty, 2013). After removing the skin, applying pressure to a spinous process also blocked locomotion, consistent with the involvement of inputs other than cutaneous in the inhibition of the spinal locomotor circuitry (Viala et al., 1978). Thus, at present, we cannot confirm with certainty the type of afferents involved in the inhibition of weight bearing and hindlimb locomotion with stimulation of the lumbar region. Additionally, a systematic investigation is required to determine the dermatomes and somatosensory afferent types with potential inhibitory actions on rhythmic activity in humans.

### Facilitation of the Spinal Locomotor CPG With Mechanical Stimulation of the Perineal Region

Stimulation of the skin of the perineal region (scrotum, vulva, base of tail and inguinal fold) facilitates hindlimb locomotion and weight support in spinal mammals. For decades, researchers have used perineal stimulation to initiate fictive locomotion in acute spinal decerebrate curarized preparations treated with clonidine or L-3,4-dihydroxyphenylalanine (L-DOPA) (Forssberg and Grillner, 1973; Pearson and Rossignol, 1991; Pearson et al., 1992; McCrea et al., 1995; Bennett et al., 1996) and to facilitate or reinforce hindlimb locomotion in chronic spinal mammals (Barbeau and Rossignol, 1987; Belanger et al., 1996; Leblond et al., 2003; Langlet et al., 2005; Hochman, 2012; Alluin et al., 2015; Harnie et al., 2019). However, the increase of spinal neuronal excitability is mediated by an undefined mechanism (Pearson and Rossignol, 1991; Harnie et al., 2019). We recently showed, in chronic spinal cats with the hindlimbs restrained, that stimulating the perineal region with vibration or pinch facilitated or triggered rhythmic locomotor-like activity (Merlet et al., 2021). In this study, a base rhythm was initiated by electrically stimulating the superficial peroneal and distal tibial nerves at 0.5 Hz. The electrical stimuli initiated flexor bursts approximately every 2 s and extensor bursts occurred spontaneously and were terminated by the next electrical stimulus. Thus, electrical stimulation initiated and entrained the rhythm. The addition of mechanical stimulation (pinch or vibration) of the perineal region significantly modulated the rhythmic activity initiated by the electrical nerve stimulation by increasing the frequency and amplitude of bursts in hindlimb muscles. It also disrupted the entrainment of the rhythm by the electrical stimuli by reducing cycle duration, possibly imposing its own entrainment. After stopping perineal stimulation, the electrical nerve stimulation resumed entraining the rhythm. Although perineal stimulation improves locomotor performance in chronic spinal cats with weak locomotor activity, it has a negligible or even detrimental effect on cats with an already robust locomotor pattern (Harnie et al., 2019). Indeed, in spinal cats with a robust hindlimb locomotion, perineal stimulation disrupts the pattern, producing exaggerated flexion or extension movements and improper left-right alternation. In other words, too much excitability to the spinal locomotor CPG is detrimental.

What type of afferent fibers mediate the facilitatory effect from the perineal region? The skin and muscles around the perineal region are innervated by the pudendal nerve inserting at the level of the second, third and fourth sacral roots (Martin et al., 1974). To our knowledge, no studies have determined the type of cutaneous fibers that mediate the excitatory effect on the spinal locomotor CPG. However, stimulating nociceptive or non-nociceptive sacral afferents is known to evoke fictive locomotor-like activity in isolated spinal cord preparations of neonatal mouse and rats (Lev-Tov et al., 2000; Strauss and Lev-Tov, 2003; Etlin et al., 2013; Mandadi et al., 2013). Thus, it is likely that low- and high-threshold afferents from the perineal region facilitate hindlimb locomotion.

## Somatosensory Feedback From Lumbar and Perineal Regions, Respectively, Increase and Decrease Reflex Gain

One approach to determine how inputs from the lumbar and perineal regions affect spinal sensorimotor circuits is to evoke reflexes while applying mechanical stimulation to the lumbar or perineal regions. One study showed that somatosensory feedback from the lumbar skin altered responses to stretch of the triceps surae muscles in decerebrate spinal cats treated with clonidine where the hindlimbs were restrained (Frigon et al., 2012b). A 2.5 s ramp-and-hold stretch evoked locomotor-like bursts in extensor and flexor muscles bilaterally that continued after the stretch. The same stretch applied while pinching the lumbar skin mainly produced a large sustained activity in the ipsilateral semitendinosus, a muscle not stretched, with no activity in the ipsilateral soleus.

**FIGURE 1:**
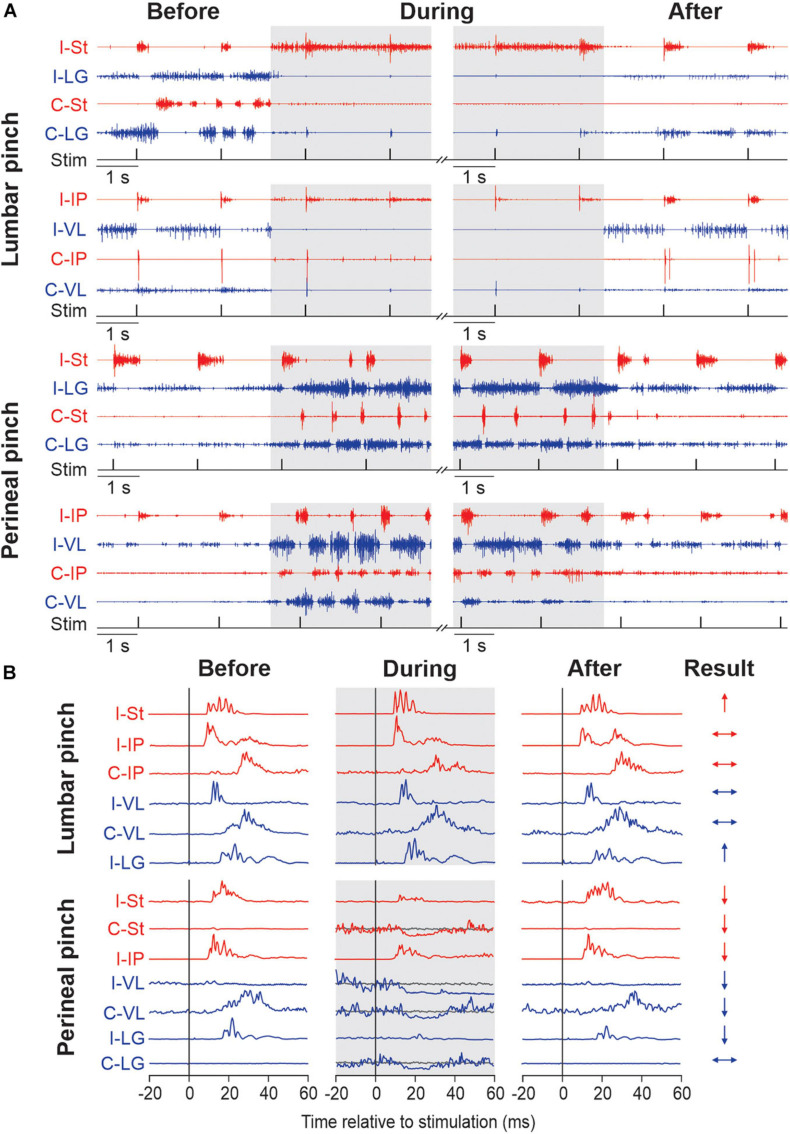
Modulation of hindlimb muscle activity and electrically evoked cutaneous reflexes when pinching the lumbar or perineal skin. **(A)** Each panel shows the electromyography (EMG) activity from the ipsilateral (I) and contralateral (C) semitendinosus (St, blue), lateralis gastrocnemius (LG, red), iliopsoas (IP, blue) and vastus lateralis (VL, red) while stimulating the superficial peroneal nerve before, during (in gray) and after pinch of the lumbar and perineal regions. The timing of the stimulation (Stim) is shown below the EMGs. Panels are from two representative cats. **(B)** Short-latency ipsilateral and contralateral reflex responses evoked by stimulating the superficial peroneal (SP) are shown for each period of the trial in selected hindlimb muscles. Waveforms are averages of 13–15 stimulations per period for a representative cat in an 80 ms window. To better visualize short-latency inhibitory responses, we superimposed averaged traces that received a stimulation (line in blue or red) with averaged traces without stimulation (line in gray). On the right, the results of reflex responses modulation of selected muscles with lumbar or perineal pinch are illustrated (↑, significant increase; ↓, significant decrease; ↔, non-significant difference in reflex responses). The figure is modified and reproduced with permission from Merlet et al. (2020, 2021).

Recently, we investigated the modulation of cutaneous reflexes from the foot by stimulating the lumbar (Merlet et al., 2020) and perineal (Merlet et al., 2021) regions in chronic spinal cats with the hindlimbs restrained. These cats did not receive a pharmacological treatment. To evoke cutaneous reflexes, we electrically stimulated (trains of 3 pulses at 300 Hz every 2 s) the superficial peroneal and distal tibial nerves at ankle level and recorded responses in ipsilateral and contralateral muscles. We showed that mechanically stimulating the lumbar region with vibration or pinch increased ipsilateral and crossed short-latency excitatory cutaneous reflex responses while perineal stimulation, in contrast, decreased reflex responses. In other words, stimulating the lumbar region inhibits locomotor and weight bearing activity but increases reflex gain while perineal stimulation produces an opposite effect, facilitation of locomotion and weight bearing while reducing reflex gain. Interestingly, perineal stimulation could also change the type of reflex response evoked by nerve stimulations, from positive to negative responses. Figure 1 illustrates the main effects of mechanically stimulating the lumbar and perineal regions on rhythmic activity and on electrically evoked cutaneous reflexes in hindlimb muscles of chronic spinal cats with the hindlimbs restrained.

## Mechanisms Involved in Modulating Spinal Locomotor CPG Activity and Cutaneous Reflexes

How do inputs from the lumbar and perineal regions inhibit or facilitate hindlimb locomotion? In contrast to stimulating the lumbar region, inputs from the perineal region do not reset the rhythm to flexion or extension. Instead, perineal inputs appear to boost the overall excitability of the network generating locomotion by increasing the magnitude of muscle activity and the cadence. Let us explain these results by considering that the spinal locomotor CPG is separated in two levels, rhythm generation and pattern formation, a concept originally proposed by Perret et al. (1988) [see McCrea and Rybak (2008) for a review and discussion]. In this organization, the rhythm generator sets the timing (i.e., the clock function) while the pattern formation layer controls motoneuron activations. As inputs from the lumbar skin often reset the rhythm to flexion, this is generally taken as evidence that signals have direct access to the rhythm generator (Hultborn et al., 1998; McCrea and Rybak, 2008; Pearson, 2008; Gossard et al., 2011; Frigon, 2012). On the other hand, inputs from the perineal region do not reset the rhythm. Instead, they increase the magnitude of muscle activity with a slight increase in cadence. Thus, inputs from the perineal region would primarily access the pattern formation level with weak to no effects on the rhythm generator, as the pattern formation level also has some rhythmogenic capabilities (McCrea and Rybak, 2008).

Where do sensory inputs from the lumbar and perineal regions project to in the spinal cord in relation to the neuronal circuits involved in locomotion? In the cat, lumbar skin afferents project to segments L4 and L5 (Kuhn, 1953). These spinal segments contain motoneuron pools that innervate hip flexors (e.g., iliopsoas and sartorius), the quadriceps, as well as hip adductors (Vanderhorst and Holstege, 1997). The mid-lumbar segments in the cat also contain critical rhythm generating elements for locomotion (Marcoux and Rossignol, 2000; Langlet et al., 2005). Thus, anatomically, inputs from the lumbar skin project directly to segments that likely contain the spinal locomotor CPG. In the cat, afferents from the perineal region enter the spinal cord more caudally, at sacral segments S1-S3 (Kuhn, 1953). One study showed that electrically stimulating the sensory pudendal or superficial perineal nerves evoked field potentials in medial parts of laminae V, VI and X at S1-S3 in the spinal cord of chloralose anesthetized or decerebrate cats (Fedirchuk et al., 1992). In cats, the rostral sacral segments contain motoneuron pools that innervate the bladder, the urethral sphincter muscle and pelvic floor muscles (Vanderhorst and Holstege, 1997). They also contain motoneurons of hindlimb muscles, such as the hamstring (e.g., semitendinosus, biceps femoris) and gluteal (e.g., gluteus medius and maximus) muscles, ankle extensors (e.g., triceps surae, plantaris) and distal muscles of the hindpaw (Vanderhorst and Holstege, 1997). Motoneurons innervating tail muscles are also located at sacral levels (Wada et al., 1990). Thus, perineal afferents also project directly to spinal segments that contain motoneurons to multiple hindlimb muscles. Although, the main core of the spinal locomotor CPG is likely at mid-lumbar segments in the cat, rhythmogenic capabilities are distributed over several spinal segments. One study showed that injecting α noradrenergic blockers from T10 to L7, the most caudal segment investigated, prevented spontaneous locomotion in decerebrate cats (Delivet-Mongrain et al., 2008), suggesting that noradrenergic mechanisms in these segments are important for rhythmogenic capabilities.

How do sacral segments interact with lumbar circuits that generate hindlimb locomotion? Lev-Tov and colleagues have investigated this question using the isolated spinal cord preparation of the neonatal rat [reviewed in Lev-Tov et al. (2010), Cherniak et al. (2014)]. The sacral spinal cord contains rhythm-generating elements that are strongly activated by somatosensory afferents and by noradrenergic and glutamatergic agonists. Interestingly, under most experimental conditions, the sacral rhythm-generating network is concurrently active with the lumbar spinal locomotor CPG. For instance, bath application of the glutamatergic agonist N-Methyl-D-aspartate (NMDA) and serotonin to the isolated neonatal rat spinal cord generates rhythmic motor bursts at lumbar and sacral levels (Cherniak et al., 2014). With spinal transection at the lumbosacral junction, the lumbar rhythm continues while the sacral rhythm stops (Cherniak et al., 2014). In contrast, with methoxamine, an α-1 noradrenergic agonist, applied to the isolated spinal cord, spinal transection at the lumbosacral junction abolishes the lumbar rhythm while the sacral rhythm continues. These results indicate powerful bi-directional interactions between rhythm-generating networks located at lumbar and sacral levels. Functionally, these interactions could help coordinate tail and hindlimb movements in certain mammals, such as rats and cats. Studies have shown that inputs from somatosensory afferents entering at sacral levels project, through crossed and uncrossed projections, directly to the lumbar locomotor CPG and indirectly via propriospinal relay neurons (Lev-Tov et al., 2000; Strauss and Lev-Tov, 2003). As stated earlier, these inputs originate from low- and high-threshold somatosensory afferents (Lev-Tov et al., 2010). Whether similar interactions between lumbar and sacral rhythm-generating networks persist in the adult spinal cord requires investigation, although rhythmic activity does occur in tail motoneurons of adult cats with a high spinal transection treated with L-DOPA and nialamide (Wada et al., 1996).

How does somatosensory feedback from the lumbar and perineal regions affect transmission in reflexes from the foot evoked by stimulating the superficial peroneal and distal tibial nerves? Cutaneous reflexes from the foot are mediated through polysynaptic pathways and project to several motor pools bilaterally via spinal interneurons intercalated between primary afferents and motoneurons (Lundberg et al., 1977; Pierrot-Deseilligny et al., 1981; Crone et al., 1987; Jankowska, 1992). In our studies, the modulation of cutaneous reflex gain with lumbar or perineal stimulation was independent of background electromyography activity preceding the sensory volley, consistent with premotoneuronal mechanisms (Merlet et al., 2020, 2021). Premotoneuronal mechanisms can include gating the transmission of interneurons within or outside the reflex pathway or by changing neurotransmitter release at the primary afferent terminal (e.g., through presynaptic inhibition). Afferents from the superficial peroneal and distal tibial nerves project to spinal segments L5 to L7 (Kuhn, 1953). Remember that lumbar skin and perineal afferents mainly project to spinal segments L4–L5 and S1–S3, respectively. It is known that cutaneous afferents upon entering the spinal cord branch out and project to a few segments rostrocaudally (Abraira and Ginty, 2013). As such, there is overlap in projections between cutaneous afferents from the lumbar and perineal regions and those traveling in the superficial peroneal and distal tibial nerves. This means that lumbar and perineal afferents can interact with local interneurons that transmit cutaneous inputs from primary afferents of the superficial peroneal and distal tibial nerves or that control presynaptic inhibition of these afferents. The increase in the gain of reflexes evoked by stimulating superficial peroneal and distal tibial nerve afferents during lumbar stimulation could involve a disinhibition of these afferents. As we observed a generalized decrease in the gain of reflexes from superficial peroneal and distal tibial nerve afferents to multiple hindlimb muscles during perineal stimulation, the simplest explanation is reduced neurotransmitter released from primary afferent terminals.

A change in the state of the spinal network, triggered by stimulating the lumbar or perineal regions, likely played an important role in modulating cutaneous reflexes from the foot. Mechanically stimulating the lumbar skin stops locomotion. Thus, the spinal network goes from a locomotor state to another state not conducive to generate locomotion. We cannot call this a ‘resting’ state because we frequently observe sustained activity in flexor muscles. It is possible that the flexor-generating portion of the spinal locomotor CPG becomes stuck in flexion. Perineal stimulation, on the other hand, increases the excitability of the spinal locomotor network. Although this is not a change in state *per se*, the locomotor network is in a more excitable state. We know that locomotion modulates cutaneous reflexes from the foot in a state-dependent manner. For example, cutaneous reflexes in spinal cats are modulated with speed (Hurteau et al., 2017) and with left-right speed differences during split-belt locomotion (Hurteau and Frigon, 2018). Studies in humans have also shown that cutaneous reflexes are modulated in a task-dependent manner (reviewed in Zehr and Stein, 1999), such as standing versus running (Duysens et al., 1993; Tax et al., 1995) or walking (Komiyama et al., 2000), and in cycling versus static contraction (Zehr et al., 2001). In parallel with observations made in cats, it has been suggested that the task-dependent cutaneous reflex modulation observed in humans is mediated in part by the spinal locomotor CPG (Brooke et al., 1997; Duysens and Van de Crommert, 1998).

It is also possible that the modulation of cutaneous reflexes is due to the activation of spinal circuits that control other functions by afferents from the lumbar and perineal regions. For example, in our spinal cats, we often observe that perineal stimulation or hindlimb locomotion triggers micturition, even with a small amount of urine in the bladder, as we thoroughly empty the bladders before experimentation. Although this is discussed in more detail later on, it is in line with other studies in chronic spinal cats that have reported that bladder contractions, induced by the opiate antagonist naloxone (Thor et al., 1983), by electrical or mechanical perigenital stimulation (Tai et al., 2006, 2008) or occurring spontaneously during micturition (Giuliani and Smith, 1987) also triggered and/or coincided with hindlimb stepping movements (Jolesz et al., 1982; Thor et al., 1983; Giuliani and Smith, 1987; Tai et al., 2006). Micturition is also a potent modulator of transmission in cutaneous reflexes to hindlimb muscles. For instance, one study showed that micturition reduced excitatory post-synaptic potentials in hindlimb motoneurons evoked by stimulating perineal or pudendal afferents in decerebrate cats (Fedirchuk et al., 1994). Studies have reported primary afferent depolarization (PAD), an indicator of presynaptic inhibition, in perineal and pudendal afferents during micturition, with no excitability changes occurring with bladder distension in the absence of micturition (Angel et al., 1994; Buss and Shefchyk, 1999). Micturition also seems to produce inhibition of interneurons interposed in the pathways from perineal and pudendal afferents to hindlimb motoneurons (Buss and Shefchyk, 2003). As illustrated by Shefchyk (2006), the central circuitry for micturition has access to various inhibitory pathways. Some of these inhibitory pathways likely influence transmission in cutaneous afferents from the foot.

**FIGURE 2:**
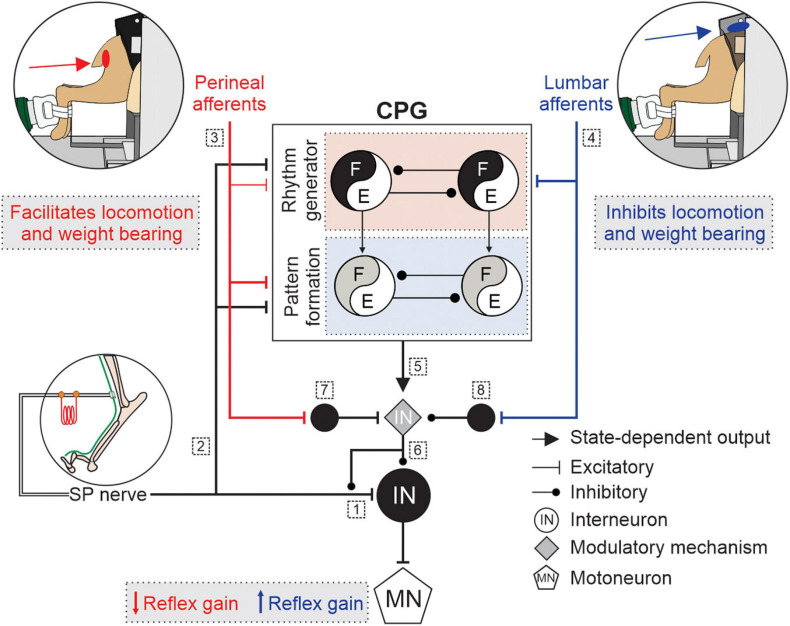
Schematic illustration of potential mechanisms modulating hindlimb locomotion and cutaneous reflexes with lumbar or perineal stimulation. Some potential mechanisms involved in the modulation of hindlimb locomotion and cutaneous reflexes, evoked by superficial peroneal (SP) nerve stimulation, with lumbar (blue) or perineal stimulation (red) are illustrated. Afferents from the SP nerve project to (1) spinal neurons and elicit reflex responses and to (2) a two-level central pattern generator (CPG) that includes rhythm generation (RG), and pattern formation (PF) levels. (3) Inputs from the perineal region primarily affect the PF level with weaker effects on the RG whereas (4) inputs from the lumbar region directly access the RG level. (5) The state of the spinal locomotor CPG controls the modulatory mechanism. (6) The modulatory mechanism controls neurotransmitter release of primary afferents from the SP nerve and the excitability of neurons within the reflex pathway. Inputs from the (7) perineal or (8) lumbar region projects to neurons that regulate the excitability of the modulatory mechanism. E, extensors; F, flexors; IN, interneurons; MN, motoneuron.

Therefore, it is likely that the spinal networks for locomotion or micturition gate the activity of neurons in reflex pathways from the superficial peroneal and distal tibial nerves to hindlimb motoneurons by changing the balance between excitation and inhibition at one or several modulation sites. To summarize, we propose that somatosensory feedback from lumbar or perineal regions, the spinal locomotor CPG and/or the spinal circuitry for micturition project to a mechanism that controls neurotransmitter release of primary afferents and the excitability of neurons within the reflex pathways, thereby increasing or decreasing reflex gain. The modulatory mechanism, which may or may not be part of the spinal locomotor CPG, could involve networks of inhibitory interneurons that regulate pre- and post synaptic inhibition. Figure 2 illustrates potential mechanism involved in the modulation of hindlimb locomotion and cutaneous reflexes, evoked by superficial peroneal nerve stimulation, with lumbar and perineal stimulation. It is, however, important to consider that the observed effects on cutaneous reflexes and hindlimb locomotion in response to stimulation of lumbar or perineal region could result from changes within the spinal cord induced by the transection and loss of supraspinal inputs.

### Changes in Reflex Pathways Following Spinal Cord Injury

After a complete SCI, sensorimotor functions and interactions in the spinal cord are dramatically altered because of the loss of descending inputs, resulting in marked modification and reorganization of synaptic transmission at the level of primary afferents, interneurons, and motoneurons (reviewed in Hultborn, 2003; Frigon and Rossignol, 2006; Rossignol and Frigon, 2011). An immediate effect of complete SCI is the loss of neuronal excitability. Indeed, brainstem pathways release potent neuromodulators, such as serotonin and noradrenaline, that adjust the excitability of spinal neurons (Hammar and Jankowska, 2003; Hammar et al., 2004; Heckman et al., 2009; Noga et al., 2009, 2011; García-Ramírez et al., 2014). These monoamines activate intrinsic membrane properties, such as persistent sodium or calcium inward currents (PICs) that generate sustained depolarizations (Heckman et al., 2003; Hultborn, 2003). Immediately after SCI, the loss of monoaminergic drive markedly decreases spinal reflexes due to the decrease in the excitability of interneurons and motoneurons (Kuhn, 1950; Conway et al., 1987; Bennett et al., 1999, 2001, 2004; Hultborn, 2003). In humans, stretch reflexes are also absent or depressed during spinal shock, immediately after SCI (Leis et al., 1996; Hultborn, 2003). As neuronal excitability returns, highlighted by the ability to generate PICs and plateau potentials (Bennett et al., 2001; Murray et al., 2010), spinal reflexes recover and can become exaggerated or disorganized (Hultborn, 2003; Frigon et al., 2011, 2012a). The same observations have been made in humans with SCI (Hiersemenzel et al., 2000; Hultborn, 2003).

Cutaneous reflexes are greatly impacted by the spinal transection because spinal interneuronal networks mediating cutaneous reflexes receive strong inhibitory projections from supraspinal structures (Schomburg, 1990) and/or because of the expansion of cutaneous receptive fields, as reported in rats (Schouenborg et al., 1992) and humans (Andersen et al., 2004). For example, stimulating cutaneous nerves during stance normally evokes short-latency inhibition in extensor muscles of intact cats (Duysens, 1977; Abraham et al., 1985; Frigon and Rossignol, 2006, Frigon and Rossignol, 2007, 2008; Frigon et al., 2009; Hurteau et al., 2018). However, after spinal transection, the same cutaneous inputs generate a short-latency excitatory response (Forssberg et al., 1975; Frigon and Rossignol, 2008). In humans, cutaneous transmission is also altered after SCI (Levin and Chapman, 1987; Fung and Barbeau, 1994; Knikou and Conway, 2001). These studies evaluated changes in transmission of cutaneous pathways by stimulating low threshold cutaneous afferents and observing modulatory effects on soleus H-reflexes (Levin and Chapman, 1987; Fung and Barbeau, 1994; Knikou and Conway, 2001). For example, Levin and Chapman (1987) reported that transmission in the pathways mediating the facilitation from the superficial peroneal nerve is depressed in complete SCI subjects compared with intact individuals. These reflex changes could be due to modifications in inhibitory influences within spinal sensorimotor circuits, such as altered presynaptic and/or reciprocal inhibition (Frigon and Rossignol, 2006, 2008). Other possible changes in reflex circuitry include the activation of latent connections, changes in synaptic strength via long−term potentiation or depression and formation of new connections through axonal sprouting (Frigon and Rossignol, 2006, 2008). Alterations in processing of spinal interneurons seems a likely mechanism in reflex changes after SCI, which would directly modify cutaneous transmission.

## Spinal Cord Injury Leads to a Loss of Functional Specificity in Response to Somatosensory Feedback

In an intact healthy system, the activation of somatosensory afferents leads to a predictable functional response. However, after SCI, the same somatosensory stimuli can lead to abnormal responses, such as occurs with the development of spasticity, broadly defined as the appearance of hyperreflexia, clonus, and muscle spasms (reviewed in Dietz, 2000; Biering-Sørensen et al., 2006; Nielsen et al., 2007). In other words, there is a loss of functional specificity.

One example showing the loss of functional specificity is with changes in simple reflex pathways, such as the stretch reflex. In a healthy system, stretching a muscle generally elicits excitatory responses in the stretched muscle and its synergists. However, after SCI, stretch of a given muscle can activate muscles throughout the legs. Frigon et al. (2011) showed that stretching the triceps surae muscles did not elicit activity in the soleus muscle, a mono-articular ankle extensor, of acute and chronic spinal-transected decerebrate cats, with variable activity in the bifunctional lateral gastrocnemius muscle, an ankle extensor and knee flexor. Instead, the stretch of the triceps surae muscles consistently evoked activity in other muscles, such as the semitendinosus (knee flexor/hip extensor), the anterior sartorius (hip flexor/knee extensor) and the tibialis anterior (ankle flexor) muscles. The same observation has been made in people with SCI. Dimitrijevic and Nathan (1967) observed a post-stretch discharge that could spread to several muscles of both legs. This loss of functional specificity between stretch-related inputs and spinal neurons likely impairs motor coordination after SCI.

The loss of functional specificity is not limited to simple reflex pathways. As stated earlier, another example demonstrating the loss of functional specificity after SCI is the concurrent activation of micturition and locomotion. In spinal cats, micturition can be triggered by having the animals step on a treadmill or by mechanically stimulating the perineal region, which also elicits stepping movements. Why does concurrent activation of micturition and hindlimb locomotion occur after spinal transection? To understand this, we must first understand how micturition is controlled. For excellent reviews on the control of micturition in animals and humans, we refer the reader to de Groat et al. (2001, 2014), Shefchyk (2002, 2006). Contraction of the smooth muscle of the bladder and the striated urethral sphincter muscle prevents micturition by closing the urethra (de Groat et al., 2001; Shefchyk, 2006). Spinal motoneurons, located at S1-S3, control the urethral sphincter muscle (Vanderhorst and Holstege, 1997). For micturition to occur, sacral parasympathetic bladder preganglionic neurons suppress sphincter muscle activity (Barrington, 1914; Sackman and Sims, 1990; Shefchyk, 2006). This opens the urethra and lets urine out of the contracting bladder. Sphincter motoneurons receive excitatory segmental sensory inputs from perineal (a branch of the pudendal nerve), pudendal and urethral afferents, as well as descending inputs from the brain (Fedirchuk et al., 1994; Buss and Shefchyk, 1999; Shefchyk, 2006). Sphincter motoneurons, in contrast to other spinal motoneurons, have weak or absent monosynaptic inputs from primary afferents and they lack functional reciprocal and recurrent inhibition (Mackel, 1979; Buss and Shefchyk, 1999; Shefchyk, 2006). Sphincter motoneurons are well suited for tonic discharge because of their high excitability and membrane input resistance (Hochman et al., 1991; Sasaki, 1991; Shefchyk, 2006). This tonic discharge maintains closure of the urethra when the bladder fills, which is most of the time. Sphincter motoneurons also display sustained activity, or PICs, which are facilitated by serotonin and noradrenaline (Paroschy and Shefchyk, 2000). This undoubtedly contributes to the tonic discharge of sphincter motoneurons, thus keeping the urethra closed.

Similar to the mesencephalic locomotor region, which elicits locomotion when electrically stimulated (Shik et al., 1966), a neuronal circuitry in the brainstem can initiate micturition without requiring somatosensory feedback, as shown with electrical stimulation of the brainstem in decerebrate cats following lumbosacral deafferentation (Shefchyk, 1989). Micturition occurs by inhibiting urethral sphincter motoneurons through the activation of a chloride conductance, as shown in decerebrate cats (Fedirchuk and Shefchyk, 1993; Shefchyk et al., 1998). This inhibition is mediated by local GABAergic and glycinergic spinal interneurons (Blok et al., 1997, 1998; Sie et al., 2001). As stated, hindlimb motoneurons receive excitatory inputs from perineal and pudendal afferents (Fedirchuk et al., 1994; Buss and Shefchyk, 1999). However, during micturition, transmission in these pathways is normally reduced or completely suppressed. Complete SCI disrupts descending pathways that interact with neuronal circuits controlling locomotion and micturition, leading to their concurrent activation. This concurrent activation suggests that bladder afferents project to the spinal locomotor CPG or that the neuronal circuits generating micturition and locomotion share common neuronal elements, or both. The abnormal activation of micturition with perineal stimulation could also result from the re-emergence of a suppressed neonatal response. Activating mechanoreceptors from the perineal region in neonatal kittens and rats, when the mother licks the perineal region of the neonate, has an excitatory effect on neurons producing bladder contractions (reviewed in de Groat et al., 2014). Similar reflexes have been identified in human infants (Boehm, 1966). This micturition reflex gradually disappears during postnatal development, becoming inhibitory. However, it re-emerges after SCI in adult mammals and humans (de Groat et al., 2014).

Two interesting models have been proposed to explain changes in spinal sensorimotor functions and interactions after SCI. The first is Wolpaw’s “Negotiated equilibrium hypothesis” (Wolpaw, 2018). In this model, brain and spinal cord plasticity interact, or negotiate, to generate new behaviors while maintaining old ones. For example, this negotiated equilibrium ensures that we do not forget to walk if we learn to dance, which likely makes use of shared spinal neuronal circuits. Each behavior results from plasticity in the brain that induces and maintains plasticity in the spinal cord. After complete SCI, however, the control normally provided by the brain is lost and the spinal cord can no longer update the brain on behavioral performance. In other words, the brain can no longer negotiate with the spinal cord to maintain neuronal properties and synaptic functions in an equilibrium to maintain innate and acquired behaviors. Without the brain to guide its plasticity, maladaptive changes occur in the spinal cord. This results in sensorimotor deficits and loss of function and specificity. With incomplete SCI, although negotiations between the brain and spinal cord continue, they are abnormal, depending on the severity of the injury and spared pathways/circuits.

The second model is Martin’s competition model. In this model descending pathways from supraspinal structures and somatosensory afferents “compete” for space in the spinal cord and control of its functions (Jiang et al., 2016, 2019). To demonstrate this, Jiang et al. (2016) investigated changes in corticospinal connectivity in the rat spinal cord with chronic electrical stimulation of proprioceptive afferents or following dorsal root sections. Stimulating proprioceptive afferents caused sprouting of their projections and withdrawal of corticospinal axons. In contrast, dorsal roots sections led to increased corticospinal connections. Thus, according to the competition model, after complete SCI, the loss of connections from supraspinal structures leads to sprouting of somatosensory afferents that fill the space left by retracting supraspinal axons. As descending inputs no longer control somatosensory afferents, this leads to maladaptive changes in spinal cord function.

Therefore, these models could explain sensorimotor deficits and loss of function and specificity observed after SCI, such as the concurrent activation of micturition and hindlimb locomotion as well as the development of spasticity.

## Functional and Clinical Considerations

What is the functional significance of the pathways from the lumbar and perineal regions to spinal networks that generate locomotion and weight support? The inhibitory effect of mechanically stimulating the lumbar region in rabbits reflects a state of “hypnotic” akinesia (Viala and Buser, 1974). Some vertebrate species have “hypnotic responses” to external stimuli (Gallup, 1974), defined as a behavioral state where the animal is immobilized and desensitized to external stimuli (Fleischmann, 1988; Pozza et al., 2008). In some mammals, including cats, immobilization can be induced by pinching or clipping the skin at certain sites, such as the neck, to facilitate the carrying of a newborn by its mother (Pozza et al., 2008). The inhibitory effect of mechanically stimulating the lumbar region could also facilitate mating behavior (Van der Horst and Holstege, 1998) or evoke a rapid transition to a crouching gait to move the body away from the stimulus (Hurteau et al., 2015). The functional purpose of the pathway from the perineal region to the spinal locomotor CPG is not clear. It could play an important survival function, such as facilitating the switch from an exploratory to an escape behavior when a predator contacts this sensitive area (Smith et al., 1988; Lev-Tov and Delvolvé, 2000; Lev-Tov et al., 2000; Whelan et al., 2000; Delvolvé et al., 2001; Bonnot et al., 2002; Strauss and Lev-Tov, 2003; Frigon and Rossignol, 2006; Cherniak et al., 2014; Merlet et al., 2021). Therefore, the spinal locomotor CPG might increase or decrease the gain of cutaneous reflexes from the foot to modulate the effects of somatosensory inputs from the lumbar or perineal region in order to adapt the animal’s behavior to task demands.

From a clinical perspective, the inhibitory mechanism from the dorsal lumbar region to spinal locomotor networks appears to have been conserved in humans (Nadeau et al., 2010), as stated earlier. Interestingly, studies have shown that transcutaneous spinal cord stimulation of the dorsal lumbar region activates the spinal locomotor network (Gorodnichev et al., 2012; Musienko et al., 2013; Gerasimenko et al., 2015). It also reduces some manifestations of spasticity in complete or incomplete SCI subjects, such as the resistance to passive stretch and the occurrence of muscle spasms evoked by cutaneous stimulation (Hofstoetter et al., 2014, 2020; Estes et al., 2017; Inanici et al., 2021). These reductions might be due to the activation of spinal circuits receiving inputs from the lumbar skin. Indeed, studies that stimulate the lumbar skin, electrically or mechanically (e.g., a harness supporting bodyweight during locomotor training), should consider the potential inhibitory mechanism from the dorsal lumbar region to spinal circuits controlling locomotion and weight support (Hurteau et al., 2015).

Surprisingly, the potential benefits of stimulating the perineal region on locomotor recovery in humans has received little attention. However, in complete or incomplete SCI subjects, dorsal penile or clitoral nerve stimulation is effective for treating neurogenic detrusor hyperactivity and increasing cystometric bladder capacity (Wheeler et al., 1992; Prévinaire et al., 1996, 1998; Kirkham et al., 2001; Dalmose et al., 2003; Hansen et al., 2005; Yoo et al., 2007; Horvath et al., 2009). Whether stimulating perineal afferents affects other sensorimotor functions remains to be investigated. Additionally, studies reported improvements in multiple aspects of urogenital and bowel functions, such as bladder capacity, following locomotor training in SCI patients (Hubscher et al., 2018; Herrity et al., 2021), suggesting a potential link between locomotor movements and other non-locomotor systems.

## Concluding Remarks and Perspectives

Somatosensory feedback is an essential component of mammalian locomotion control. Although most studies have focused on feedback from the limbs, inputs from the lumbar and perineal regions also exert powerful effects on weight support and hindlimb locomotion. However, despite the potent effects of somatosensory feedback from the lumbar or perineal regions on weight support and locomotor activity in mammals with complete SCI, this area of research as a therapeutic approach is largely unexplored. We have attempted to summarize how stimulation of the lumbar or perineal region modulates the spinal locomotor CPG and the excitability of reflex circuits activated by foot afferents, as well as the potential mechanisms involved. Overall, further investigations are required to increase our knowledge as to how somatosensory feedback from the lumbar and perineal regions interacts with spinal sensorimotor circuits and to determine the benefits of stimulating these regions in pathological conditions. This knowledge could greatly benefit humans with sensorimotor disorders.

## Author Contributions

JH and AF conceived and designed the research. JH performed the experiments. AM analyzed the data and performed the statistical analysis. AM, JH, and AF interpreted the results, prepared the figures, edited and revised the manuscript, and approved the final version. All authors contributed to the article and approved the submitted version.

## Conflict of Interest

The authors declare that the research was conducted in the absence of any commercial or financial relationships that could be construed as a potential conflict of interest.

## Publisher’s Note

All claims expressed in this article are solely those of the authors and do not necessarily represent those of their affiliated organizations, or those of the publisher, the editors and the reviewers. Any product that may be evaluated in this article, or claim that may be made by its manufacturer, is not guaranteed or endorsed by the publisher.

## References

[B1] Abraham L. D. Marks W. B. Loeb G. E. (1985). The distal hindlimb musculature of the cat: cutaneous reflexes during locomotion. *Exp. Brain Res.* 58 594–603. 10.1007/BF00235875 4007097

[B2] Abraira V. E. Ginty D. D. (2013). The sensory neurons of touch. *Neuron* 79 618–639. 10.1016/j.neuron.2013.07.051 23972592 PMC3811145

[B3] Alluin O. Delivet-Mongrain H. Rossignol S. (2015). Inducing hindlimb locomotor recovery in adult rat after complete thoracic spinal cord section using repeated treadmill training with perineal stimulation only. *J. Neurophysiol.* 114 1931–1946. 10.1152/jn.00416.2015 26203108 PMC4579296

[B4] Andersen O. K. Finnerup N. B. Spaich E. G. Jensen T. S. Arendt-Nielsen L. (2004). Expansion of nociceptive withdrawal reflex receptive fields in spinal cord injured humans. *Clin. Neurophysiol.* 115 2798–2810. 10.1016/j.clinph.2004.07.003 15546788

[B5] Angel M. J. Fyda D. McCrea D. A. Shefchyk S. J. (1994). Primary afferent depolarization of cat pudendal afferents during micturition and segmental afferent stimulation. *J. Physiol.* 479 451–461. 10.1113/jphysiol.1994.sp020309 7837101 PMC1155763

[B6] Barbeau H. Rossignol S. (1987). Recovery of locomotion after chronic spinalization in the adult cat. *Brain Res.* 412 84–95. 10.1016/0006-8993(87)91442-9 3607464

[B7] Barrington F. J. F. (1914). The nervous mechanism of micturition. *Exp. Physiol.* 8 33–71. 10.1113/expphysiol.1914.sp000171

[B8] Belanger M. Drew T. Provencher J. Rossignol S. (1996). A comparison of treadmill locomotion in adult cats before and after spinal transection. *J. Neurophysiol.* 76 471–491. 10.1152/jn.1996.76.1.471 8836238

[B9] Bennett D. J. De Serres S. J. Stein R. B. (1996). Gain of the triceps surae stretch reflex in decerebrate and spinal cats during postural and locomotor activities. *J. Physiol.* 496 837–850. 10.1113/jphysiol.1996.sp021731 8930848 PMC1160868

[B10] Bennett D. J. Gorassini M. Fouad K. Sanelli L. Han Y. Cheng J. (1999). Spasticity in rats with sacral spinal cord injury. *J. Neurotrauma* 16 69–84. 10.1089/neu.1999.16.69 9989467

[B11] Bennett D. J. Li Y. Siu M. (2001). Plateau potentials in sacrocaudal motoneurons of chronic spinal rats, recorded *in vitro*. *J. Neurophysiol.* 86 1955–1971. 10.1152/jn.2001.86.4.1955 11600653

[B12] Bennett D. J. Sanelli L. Cooke C. L. Harvey P. J. Gorassini M. A. (2004). Spastic long-lasting reflexes in the awake rat after sacral spinal cord injury. *J. Neurophysiol.* 91 2247–2258. 10.1152/jn.00946.2003 15069102

[B13] Biering-Sørensen F. Nielsen J. B. Klinge K. (2006). Spasticity-assessment: a review. *Spinal Cord* 44 708–722. 10.1038/sj.sc.3101928 16636687

[B14] Blok B. F. M. de Weerd H. Holstege G. (1997). The pontine micturition center projects to sacral cord GABA immunoreactive neurons in the cat. *Neurosci. Lett.* 233 109–112. 10.1016/S0304-3940(97)00644-7 9350844

[B15] Blok B. F. M. van Maarseveen J. T. P. W. Holstege G. (1998). Electrical stimulation of the sacral dorsal gray commissure evokes relaxation of the external urethral sphincter in the cat. *Neurosci. Lett.* 249 68–70. 10.1016/S0304-3940(98)00382-6 9672391

[B16] Boehm J. J. (1966). Bacteriology of “Midstream Catch”, Urines: studies in newborn infants. *Am. J. Dis. Child.* 111 366–369. 10.1001/archpedi.1966.02090070064007 5324535

[B17] Bonnot A. Whelan P. J. Mentis G. Z. O’Donovan M. J. (2002). Spatiotemporal pattern of motoneuron activation in the rostral lumbar and the sacral segments during locomotor-like activity in the neonatal mouse spinal cord. *J. Neurosci.* 22 RC203–RC203. 10.1523/JNEUROSCI.22-03-j0001.2002 11826149 PMC6758517

[B18] Brooke J. D. Cheng J. Collins D. F. Mcilroy W. E. Misiaszek J. E. Staines W. R. (1997). Sensori afferent conditioning with leg movement: gain control in spinal reflex and ascending paths. *Prog. Neurobiol.* 51 393–421. 10.1016/S0301-0082(96)00061-5 9106899

[B19] Brooke J. D. Misiaszek J. E. Cheng J. (1993). Locomotor-like rotation of either hip or knee inhibits Soleus H reflexes in humans. *Somatosens. Mot. Res.* 10 357–364. 10.3109/08990229309028843 8310778

[B20] Buss R. R. Shefchyk S. J. (1999). Excitability changes in sacral afferents innervating the urethra, perineum and hindlimb skin of the cat during micturition. *J. Physiol.* 514 593–607. 10.1111/j.1469-7793.1999.593ae.x 9852338 PMC2269077

[B21] Buss R. R. Shefchyk S. J. (2003). Sacral dorsal horn neurone activity during micturition in the cat. *J. Physiol.* 551 387–396. 10.1113/jphysiol.2003.041996 12815177 PMC2343146

[B22] Chapman C. E. Sullivan S. J. Pompura J. Arsenault A. B. (1991). Changes in hip position modulate soleus H-reflex excitability in man. *Electromyogr. Clin. Neurophysiol.* 31 131–143. 2049989

[B23] Cherniak M. Etlin A. Strauss I. Anglister L. Lev-Tov A. (2014). The sacral networks and neural pathways used to elicit lumbar motor rhythm in the rodent spinal cord. *Front. Neural Circuits* 8:143. 10.3389/fncir.2014.00143 25520624 PMC4253665

[B24] Conway B. A. Hultborn H. Kiehn O. (1987). Proprioceptive input resets central locomotor rhythm in the spinal cat. *Exp. Brain Res.* 68 643–656. 10.1007/BF00249807 3691733

[B25] Crone C. Hultborn H. Jespersen B. Nielsen J. (1987). Reciprocal Ia inhibition between ankle flexors and extensors in man. *J. Physiol.* 389 163–185. 10.1113/jphysiol.1987.sp016652 3681725 PMC1192076

[B26] Dalmose A. L. Rijkhoff N. J. M. Kirkeby H. J. Nohr M. Sinkjaer T. Djurhuus J. C. (2003). Conditional stimulatzion of the dorsal penile/clitoral nerve may increase cystometric capacity in patients with spinal cord injury. *Neurourol. Urodyn.* 22 130–137. 10.1002/nau.10031 12579630

[B27] de Groat W. Fraser M. Yoshiyama M. Smerin S. Tai C. Chancellor M. (2001). Neural control of the urethra. *Scand. J. Urol. Nephrol.* 35 35–43. 10.1080/003655901750174872 11409613

[B28] de Groat W. C. Griffiths D. Yoshimura N. (2014). “Neural control of the lower urinary tract,” in *Comprehensive Physiology*, ed. Terjung R. (Hoboken, NJ: John Wiley & Sons, Inc), 327–396. 10.1002/cphy.c130056 PMC4480926 25589273

[B29] Delivet-Mongrain H. Leblond H. Rossignol S. (2008). Effects of localized intraspinal injections of a noradrenergic blocker on locomotion of high decerebrate cats. *J. Neurophysiol.* 100 907–921. 10.1152/jn.90454.2008 18550723

[B30] Delvolvé I. Gabbay H. Lev-Tov A. (2001). The motor output and behavior produced by rhythmogenic sacrocaudal networks in spinal cords of neonatal rats. *J. Neurophysiol.* 85 2100–2110. 10.1152/jn.2001.85.5.2100 11353026

[B31] Dietz V. (2000). Spastic movement disorder. *Spinal Cord* 38 389–393. 10.1038/sj.sc.3101030 10962597

[B32] Dietz V. (2002). Proprioception and locomotor disorders. *Nat. Rev. Neurosci.* 3 781–790. 10.1038/nrn939 12360322

[B33] Dimitrijevic M. R. Nathan P. W. (1967). Studies of spasticity in man: 2, Analysis of stretch reflexes in spasticity. *Brain* 90 333–358. 10.1093/brain/90.2.333 6028252

[B34] Duysens J. (1977). Reflex control of locomotion as revealed by stimulation of cutaneous afferents in spontaneously walking premammillary cats. *J. Neurophysiol.* 40 737–751. 10.1152/jn.1977.40.4.737 886369

[B35] Duysens J. Clarac F. Cruse H. (2000). Load-regulating mechanisms in gait and posture: comparative aspects. *Physiol. Rev.* 80 83–133. 10.1152/physrev.2000.80.1.83 10617766

[B36] Duysens J. Tax A. A. M. Trippel M. Dietz V. (1993). Increased amplitude of cutaneous reflexes during human running as compared to standing. *Brain Res.* 613 230–238. 10.1016/0006-8993(93)90903-Z 8186969

[B37] Duysens J. Van de Crommert H. W. A. A. (1998). Neural control of locomotion; Part 1: the central pattern generator from cats to humans. *Gait Posture* 7 131–141. 10.1016/S0966-6362(97)00042-8 10200383

[B38] Estes S. P. Iddings J. A. Field-Fote E. C. (2017). Priming neural circuits to modulate spinal reflex excitability. *Front. Neurol.* 8:17. 10.3389/fneur.2017.00017 28217104 PMC5289977

[B39] Etlin A. Finkel E. Mor Y. O’Donovan M. J. Anglister L. Lev-Tov A. (2013). Characterization of sacral interneurons that mediate activation of locomotor pattern generators by sacrocaudal afferent input. *J. Neurosci.* 33 734–747. 10.1523/JNEUROSCI.4390-12.2013 23303951 PMC6704898

[B40] Fedirchuk B. Downie J. Shefchyk S. (1994). Reduction of perineal evoked excitatory postsynaptic potentials in cat lumbar and sacral motoneurons during micturition. *J. Neurosci.* 14 6153–6159. 10.1523/JNEUROSCI.14-10-06153.1994 7931569 PMC6576969

[B41] Fedirchuk B. Shefchyk S. (1993). Membrane potential changes in sphincter motoneurons during micturition in the decerebrate cat. *J. Neurosci.* 13 3090–3094. 10.1523/JNEUROSCI.13-07-03090.1993 8331386 PMC6576663

[B42] Fedirchuk B. Song L. Downie J. W. Shefchyk S. J. (1992). Spinal distribution of extracellular field potentials generated by electrical stimulation of pudendal and perineal afferents in the cat. *Exp. Brain Res.* 89 517–520. 10.1007/BF00229876 1644117

[B43] Fleischmann A. (1988). Clip-induced analgesia and immobility in the mouse: pharmacological characterization. *Neuropharmacology* 27 641–648. 10.1016/0028-3908(88)90187-6 3419547

[B44] Forssberg H. Grillner S. (1973). The locomotion of the acute spinal cat injected with clonidine iv. *Brain Res.* 50 184–186. 10.1016/0006-8993(73)90606-9 4690545

[B45] Forssberg H. Grillner S. Rossignol S. (1975). Phase dependent reflex reversal during walking in chronic spinal cats. *Brain Res.* 85 103–107. 10.1016/0006-8993(75)91013-6 1109686

[B46] Frigon A. (2012). Central pattern generators of the mammalian spinal cord. *Neuroscientist* 18 56–69. 10.1177/1073858410396101 21518815

[B47] Frigon A. (2017). The neural control of interlimb coordination during mammalian locomotion. *J. Neurophysiol.* 117 2224–2241. 10.1152/jn.00978.2016 28298308 PMC5454475

[B48] Frigon A. Barrière G. Leblond H. Rossignol S. (2009). Asymmetric changes in cutaneous reflexes after a partial spinal lesion and retention following spinalization during locomotion in the cat. *J. Neurophysiol.* 102 2667–2680. 10.1152/jn.00572.2009 19726726

[B49] Frigon A. Desrochers É. Thibaudier Y. Hurteau M.-F. Dambreville C. (2017). Left-right coordination from simple to extreme conditions during split-belt locomotion in the chronic spinal adult cat: left-right coordination during locomotion. *J. Physiol.* 595 341–361. 10.1113/JP272740 27426732 PMC5199742

[B50] Frigon A. Johnson M. D. Heckman C. J. (2011). Altered activation patterns by triceps surae stretch reflex pathways in acute and chronic spinal cord injury. *J. Neurophysiol.* 106 1669–1678. 10.1152/jn.00504.2011 21734111 PMC3191838

[B51] Frigon A. Johnson M. D. Heckman C. J. (2012a). Differential modulation of crossed and uncrossed reflex pathways by clonidine in adult cats following complete spinal cord injury: clonidine alters reflex pathways after spinalization. *J. Physiol.* 590 973–989. 10.1113/jphysiol.2011.222208 22219338 PMC3381322

[B52] Frigon A. Rossignol S. (2008). Adaptive changes of the locomotor pattern and cutaneous reflexes during locomotion studied in the same cats before and after spinalization: changes in reflexes during locomotion after spinalization. *J. Physiol.* 586 2927–2945. 10.1113/jphysiol.2008.152488 18420704 PMC2517203

[B53] Frigon A. Rossignol S. (2007). Plasticity of reflexes from the foot during locomotion after denervating ankle extensors in intact cats. *J. Neurophysiol.* 98 2122–2132. 10.1152/jn.00490.2007 17652411

[B54] Frigon A. Rossignol S. Frigon A. Rossignol S. (2006). Functional plasticity following spinal cord lesions. *Prog. Brain Res.* 157 231–260. 10.1016/S0079-6123(06)57016-5 17167915

[B55] Frigon A. Sirois J. Gossard J.-P. (2010). Effects of ankle and hip muscle afferent inputs on rhythm generation during fictive locomotion. *J. Neurophysiol.* 103 1591–1605. 10.1152/jn.01028.2009 20089809

[B56] Frigon A. Thibaudier Y. Johnson M. D. Heckman C. J. Hurteau M.-F. (2012b). Cutaneous inputs from the back abolish locomotor-like activity and reduce spastic-like activity in the adult cat following complete spinal cord injury. *Exp. Neurol.* 235 588–598. 10.1016/j.expneurol.2012.03.013 22487200 PMC3345060

[B57] Fung J. Barbeau H. (1994). Effects of conditioning cutaneomuscular stimulation on the soleus H-reflex in normal and spastic paretic subjects during walking and standing. *J. Neurophysiol.* 72 2090–2104. 10.1152/jn.1994.72.5.2090 7884446

[B58] Gallup G. G. (1974). Animal hypnosis: factual status of a fictional concept. *Psychol. Bull.* 81 836–853. 10.1037/h0037227 4612575

[B59] García-Ramírez D. L. Calvo J. R. Hochman S. Quevedo J. N. (2014). Serotonin, dopamine and noradrenaline adjust actions of myelinated afferents via modulation of presynaptic inhibition in the mouse spinal cord. *PLoS One* 9:e89999. 10.1371/journal.pone.0089999 24587177 PMC3938568

[B60] Gerasimenko Y. Gorodnichev R. Puhov A. Moshonkina T. Savochin A. Selionov V. (2015). Initiation and modulation of locomotor circuitry output with multisite transcutaneous electrical stimulation of the spinal cord in noninjured humans. *J. Neurophysiol.* 113 834–842. 10.1152/jn.00609.2014 25376784

[B61] Giuliani C. Smith J. (1987). Stepping behaviors in chronic spinal cats with one hindlimb deafferented. *J. Neurosci.* 7 2537–2546. 3612253 PMC6568957

[B62] Gorodnichev R. M. Pivovarova E. A. Pukhov A. Moiseev S. A. Savokhin A. A. Moshonkina T. R. (2012). [Transcutaneous electrical stimulation of the spinal cord: non-invasive tool for activation of locomotor circuitry in human]. *Fiziol. Cheloveka* 38 46–56. 22679796

[B63] Gossard J.-P. Brownstone R. M. Barajon I. Hultborn H. (1994). Transmission in a locomotor-related group Ib pathway from hindlimb extensor muscles in the cat. *Exp. Brain Res.* 98 213–228. 10.1007/BF00228410 8050508

[B64] Gossard J.-P. Sirois J. Noué P. Côté M.-P. Ménard A. Leblond H. (2011). The spinal generation of phases and cycle duration. *Prog. Brain Res.* 188 15–29. 10.1016/B978-0-444-53825-3.00007-3 21333800

[B65] Grillner S. (1981). “Control of locomotion in bipeds, tetrapods, and fish,” in *Handbook of Physiology, Section 1, The Nervous System, Vol. 2, Motor Control*, ed. Brooks V. B. (Bethesda: American Physiological Society), 1179–1236. 10.1002/cphy.cp010226

[B66] Grillner S. El Manira A. (2020). Current principles of motor control, with special reference to vertebrate locomotion. *Physiol. Rev.* 100 271–320. 10.1152/physrev.00015.2019 31512990

[B67] Guertin P. Angel M. J. Perreault M. C. McCrea D. A. (1995). Ankle extensor group I afferents excite extensors throughout the hindlimb during fictive locomotion in the cat. *J. Physiol.* 487 197–209. 10.1113/jphysiol.1995.sp020871 7473249 PMC1156609

[B68] Hammar I. Bannatyne B. A. Maxwell D. J. Edgley S. A. Jankowska E. (2004). The actions of monoamines and distribution of noradrenergic and serotoninergic contacts on different subpopulations of commissural interneurons in the cat spinal cord. *Eur. J. Neurosci.* 19 1305–1316. 10.1111/j.1460-9568.2004.03239.x 15016088 PMC1971244

[B69] Hammar I. Jankowska E. (2003). Modulatory effects of α 1-, α 2-, and β-receptor agonists on feline spinal interneurons with monosynaptic input from group i muscle afferents. *J. Neurosci.* 23 332–338. 10.1523/JNEUROSCI.23-01-00332.2003 12514232 PMC1890035

[B70] Hansen J. Media S. Nøhr M. Biering-Sørensen F. Sinkjaer T. Rijkhoff N. J. M. (2005). Treatment of neurogenic detrusor overactivity in spinal cord injured patients by conditional electrical stimulation. *J. Urol.* 173 2035–2039. 10.1097/01.ju.0000158160.11083.1b 15879820

[B71] Harnie J. Côté-Sarrazin C. Hurteau M.-F. Desrochers E. Doelman A. Amhis N. (2018). The modulation of locomotor speed is maintained following partial denervation of ankle extensors in spinal cats. *J. Neurophysiol.* 120 1274–1285. 10.1152/jn.00812.2017 29897865 PMC6171056

[B72] Harnie J. Doelman A. de Vette E. Audet J. Desrochers E. Gaudreault N. (2019). The recovery of standing and locomotion after spinal cord injury does not require task-specific training. *eLife* 8:e50134. 10.7554/eLife.50134 31825306 PMC6924957

[B73] Harnie J. Audet J. Klishko A. N. Doelman A. Prilutsky B. I. Frigon A. (2021). The spinal control of backward locomotion. *J. Neurosci.* 41 630–647. 10.1523/JNEUROSCI.0816-20.2020 33239399 PMC7842752

[B74] Heckman C. J. Lee R. H. Brownstone R. M. (2003). Hyperexcitable dendrites in motoneurons and their neuromodulatory control during motor behavior. *Trends Neurosci.* 26 688–695. 10.1016/j.tins.2003.10.002 14624854

[B75] Heckman C. J. Mottram C. Quinlan K. Theiss R. Schuster J. (2009). Motoneuron excitability: the importance of neuromodulatory inputs. *Clin. Neurophysiol.* 120 2040–2054. 10.1016/j.clinph.2009.08.009 19783207 PMC7312725

[B76] Herrity A. N. Aslan S. C. Ugiliweneza B. Mohamed A. Z. Hubscher C. H. Harkema S. J. (2021). Improvements in bladder function following activity-based recovery training with epidural stimulation after chronic spinal cord injury. *Front. Syst. Neurosci.* 14:614691. 10.3389/fnsys.2020.614691 33469421 PMC7813989

[B77] Hiersemenzel L.-P. Curt A. Dietz V. (2000). From spinal shock to spasticity: neuronal adaptations to a spinal cord injury. *Neurology* 54 1574–1582. 10.1212/WNL.54.8.1574 10762496

[B78] Higgin D. Krupka A. Maghsoudi O. H. Klishko A. N. Nichols T. R. Lyle M. A. (2020). Adaptation to slope in locomotor-trained spinal cats with intact and self-reinnervated lateral gastrocnemius and soleus muscles. *J. Neurophysiol.* 123 70–89. 10.1152/jn.00018.2019 31693435 PMC6985865

[B79] Hochman S. (2012). Enabling techniques for *in vitro* studies on mammalian spinal locomotor mechanisms. *Front. Biosci.* 17:2158. 10.2741/4043 22652770 PMC7001871

[B80] Hochman S. Fedirchuk B. Shefchyk S. J. (1991). Membrane electrical properties of external urethral and external anal sphincter somatic motoneurons in the decerebrate cat. *Neurosci. Lett.* 127 87–90. 10.1016/0304-3940(91)90901-5 1881623

[B81] Hofstoetter U. S. Freundl B. Danner S. M. Krenn M. J. Mayr W. Binder H. (2020). Transcutaneous spinal cord stimulation induces temporary attenuation of spasticity in individuals with spinal cord injury. *J. Neurotrauma* 37 481–493. 10.1089/neu.2019.6588 31333064

[B82] Hofstoetter U. S. McKay W. B. Tansey K. E. Mayr W. Kern H. Minassian K. (2014). Modification of spasticity by transcutaneous spinal cord stimulation in individuals with incomplete spinal cord injury. *J. Spinal Cord Med.* 37 202–211. 10.1179/2045772313Y.0000000149 24090290 PMC4066429

[B83] Horvath E. E. Yoo P. B. Amundsen C. L. Webster G. D. Grill W. M. (2009). Conditional and continuous electrical stimulation increase cystometric capacity in persons with spinal cord injury. *Neurourol. Urodyn.* 29 401–407. 10.1002/nau.20766 19634166 PMC3109497

[B84] Hubscher C. H. Herrity A. N. Williams C. S. Montgomery L. R. Willhite A. M. Angeli C. A. (2018). Improvements in bladder, bowel and sexual outcomes following task-specific locomotor training in human spinal cord injury. *PLoS One* 13:e0190998. 10.1371/journal.pone.0190998 29385166 PMC5791974

[B85] Hultborn H. (2003). Changes in neuronal properties and spinal reflexes during development of spasticity following spinal cord lesions and stroke: studies in animal models and patients. *J. Rehabil. Med.* 35 46–55. 10.1080/16501960310010142 12817657

[B86] Hultborn H. Conway B. A. Gossard J.-P. Brownstone R. Fedirchuk B. Schomburg E. D. (1998). How do we approach the locomotor network in the mammalian spinal cord? *Ann. N. Y. Acad. Sci.* 860 70–82. 10.1111/j.1749-6632.1998.tb09039.x 9928302

[B87] Hurteau M.-F. Frigon A. (2018). A spinal mechanism related to left–right symmetry reduces cutaneous reflex modulation independently of speed during split-belt locomotion. *J. Neurosci.* 38 10314–10328. 10.1523/JNEUROSCI.1082-18.2018 30315129 PMC6596212

[B88] Hurteau M.-F. Thibaudier Y. Dambreville C. Chraibi A. Desrochers E. Telonio A. (2017). Nonlinear modulation of cutaneous reflexes with increasing speed of locomotion in spinal cats. *J. Neurosci.* 37 3896–3912. 10.1523/JNEUROSCI.3042-16.2017 28292829 PMC6596719

[B89] Hurteau M.-F. Thibaudier Y. Dambreville C. Danner S. M. Rybak I. A. Frigon A. (2018). Intralimb and interlimb cutaneous reflexes during locomotion in the intact cat. *J. Neurosci.* 38 4104–4122. 10.1523/JNEUROSCI.3288-17.2018 29563181 PMC5963849

[B90] Hurteau M.-F. Thibaudier Y. Dambreville C. Desaulniers C. Frigon A. (2015). Effect of stimulating the lumbar skin caudal to a complete spinal cord injury on hindlimb locomotion. *J. Neurophysiol.* 113 669–676. 10.1152/jn.00739.2014 25339715

[B91] Inanici F. Brighton L. N. Samejima S. Hofstetter C. P. Moritz C. T. (2021). Transcutaneous spinal cord stimulation restores hand and arm function after spinal cord injury. *IEEE Trans. Neural Syst. Rehabil. Eng.* 29 310–319. 10.1109/TNSRE.2021.3049133 33400652

[B92] Jankowska E. (1992). Interneuronal relay in spinal pathways from proprioceptors. *Prog. Neurobiol.* 38 335–378. 10.1016/0301-0082(92)90024-9 1315446

[B93] Jiang Y.-Q. Armada K. Martin J. H. (2019). Neuronal activity and microglial activation support corticospinal tract and proprioceptive afferent sprouting in spinal circuits after a corticospinal system lesion. *Exp. Neurol.* 321 113015. 10.1016/j.expneurol.2019.113015 31326353 PMC6856874

[B94] Jiang Y.-Q. Zaaimi B. Martin J. H. (2016). Competition with primary sensory afferents drives remodeling of corticospinal axons in mature spinal motor circuits. *J. Neurosci.* 36 193–203. 10.1523/JNEUROSCI.3441-15.2016 26740661 PMC4701960

[B95] Jolesz F. Cheng-Tao X. Ruenzel P. Henneman E. (1982). Flexor reflex control of the external sphincter of the urethra in paraplegia. *Science* 216 1243–1245. 10.1126/science.7200635 7200635

[B96] Kiehn O. (2016). Decoding the organization of spinal circuits that control locomotion. *Nat. Rev. Neurosci.* 17 224–238. 10.1038/nrn.2016.9 26935168 PMC4844028

[B97] Kirkham A. Shah N. Knight S. Shah P. Craggs M. (2001). The acute effects of continuous and conditional neuromodulation on the bladder in spinal cord injury. *Spinal Cord* 39 420–428. 10.1038/sj.sc.3101177 11512072

[B98] Knikou M. Conway B. A. (2001). Modulation of soleus H-reflex following ipsilateral mechanical loading of the sole of the foot in normal and complete spinal cord injured humans. *Neurosci. Lett.* 303 107–110. 10.1016/S0304-3940(01)01718-9 11311504

[B99] Knikou M. Rymer W. Z. (2002a). Effects of changes in hip joint angle on H-reflex excitability in humans. *Exp. Brain Res.* 143 149–159. 10.1007/s00221-001-0978-4 11880891

[B100] Knikou M. Rymer W. Z. (2002b). Hip angle induced modulation of H reflex amplitude, latency and duration in spinal cord injured humans. *Clin. Neurophysiol.* 113 1698–1708. 10.1016/S1388-2457(02)00285-7 12417222

[B101] Komiyama T. Zehr E. P. Stein R. B. (2000). Absence of nerve specificity in human cutaneous reflexes during standing. *Exp. Brain Res.* 133 267–272. 10.1007/s002210000411 10968228

[B102] Kuczynski V. Telonio A. Thibaudier Y. Hurteau M.-F. Dambreville C. Desrochers E. (2017). Lack of adaptation during prolonged split-belt locomotion in the intact and spinal cat: lack of adaptation during split-belt locomotion in the cat. *J. Physiol.* 595 5987–6006. 10.1113/JP274518 28643899 PMC5577523

[B103] Kuhn R. A. (1950). Functional capacity of the isolated human spinal cord. *Brain* 73 1–51. 10.1093/brain/73.1.1 15420313

[B104] Kuhn R. A. (1953). Organization of tactile dermatomes in cat and monkey. *J. Neurophysiol.* 16 169–182. 10.1152/jn.1953.16.2.169 13035476

[B105] Lam T. Pearson K. G. (2001). Proprioceptive modulation of hip flexor activity during the swing phase of locomotion in decerebrate cats. *J. Neurophysiol.* 86 1321–1332. 10.1152/jn.2001.86.3.1321 11535680

[B106] Langlet C. Leblond H. Rossignol S. (2005). Mid-lumbar segments are needed for the expression of locomotion in chronic spinal cats. *J. Neurophysiol.* 93 2474–2488. 10.1152/jn.00909.2004 15647400

[B107] Leblond H. L’Espérance M. Orsal D. Rossignol S. (2003). Treadmill locomotion in the intact and spinal mouse. *J. Neurosci.* 23 11411–11419. 10.1523/JNEUROSCI.23-36-11411.2003 14673005 PMC6740531

[B108] Leis A. A. Kronenberg M. F. Stetkarova I. Paske W. C. Stokic D. S. (1996). Spinal motoneuron excitability after acute spinal cord injury in humans. *Neurology* 47 231–237. 10.1212/WNL.47.1.231 8710084

[B109] Levin M. Chapman C. E. (1987). Inhibitory and facilitatory effects from the peroneal nerve onto the soleus H-reflex in normal and spinal man. *Electroencephalogr. Clin. Neurophysiol.* 67 468–478. 10.1016/0013-4694(87)90011-3 2444416

[B110] Lev-Tov A. Delvolvé I. (2000). Pattern generation in non-limb moving segments of the mammalian spinal cord. *Brain Res. Bull.* 53 671–675. 10.1016/S0361-9230(00)00400-7 11165802

[B111] Lev-Tov A. Delvolvé I. Kremer E. (2000). Sacrocaudal afferents induce rhythmic efferent bursting in isolated spinal cords of neonatal rats. *J. Neurophysiol.* 83 888–894. 10.1152/jn.2000.83.2.888 10669502

[B112] Lev-Tov A. Etlin A. Blivis D. (2010). Sensory-induced activation of pattern generators in the absence of supraspinal control: sensory-evoked motor rhythmic patterns. *Ann. N. Y. Acad. Sci.* 1198 54–62. 10.1111/j.1749-6632.2009.05424.x 20536920

[B113] Lundberg A. Malmgren K. Schomburg E. D. (1977). Cutaneous facilitation of transmission in reflex pathways from Ib afferents to motoneurones. *J. Physiol.* 265 763–780. 10.1113/jphysiol.1977.sp011742 192879 PMC1307846

[B114] Mackel R. (1979). Segmental and descending control of the external urethral and anal sphincters in the cat. *J. Physiol.* 294 105–122. 10.1113/jphysiol.1979.sp012918 512936 PMC1280545

[B115] Mandadi S. Hong P. Tran M. A. Bráz J. M. Colarusso P. Basbaum A. I. (2013). Identification of multisegmental nociceptive afferents that modulate locomotor circuits in the neonatal mouse spinal cord: long-range nociceptive collaterals. *J. Comp. Neurol.* 521 2870–2887. 10.1002/cne.23321 23436436

[B116] Marcoux J. Rossignol S. (2000). Initiating or blocking locomotion in spinal cats by applying noradrenergic drugs to restricted lumbar spinal segments. *J. Neurosci.* 20 8577–8585. 10.1523/JNEUROSCI.20-22-08577.2000 11069966 PMC6773188

[B117] Martin W. D. Fletcher T. F. Bradley W. E. (1974). Innervation of feline perineal musculature. *Anat. Rec.* 180 15–29. 10.1002/ar.1091800104 4278149

[B118] McCrea D. A. Rybak I. A. (2008). Organization of mammalian locomotor rhythm and pattern generation. *Brain Res. Rev.* 57 134–146. 10.1016/j.brainresrev.2007.08.006 17936363 PMC2214837

[B119] McCrea D. A. Shefchyk S. J. Stephens M. J. Pearson K. G. (1995). Disynaptic group I excitation of synergist ankle extensor motoneurones during fictive locomotion in the cat. *J. Physiol.* 487 527–539. 10.1113/jphysiol.1995.sp020897 8558481 PMC1156590

[B120] Merlet A. N. Harnie J. Macovei M. Doelman A. Gaudreault N. Frigon A. (2020). Mechanically stimulating the lumbar region inhibits locomotor-like activity and increases the gain of cutaneous reflexes from the paws in spinal cats. *J. Neurophysiol.* 123 1026–1041. 10.1152/jn.00747.2019 32049598

[B121] Merlet A. N. Harnie J. Macovei M. Doelman A. Gaudreault N. Frigon A. (2021). Cutaneous inputs from perineal region facilitate spinal locomotor activity and modulate cutaneous reflexes from the foot in spinal cats. *J. Neurosci. Res.* 99 1448–1473. 10.1002/jnr.24791 33527519

[B122] Murray K. C. Nakae A. Stephens M. J. Rank M. D’Amico J. Harvey P. J. (2010). Recovery of motoneuron and locomotor function after spinal cord injury depends on constitutive activity in 5-HT2C receptors. *Nat. Med.* 16 694–700. 10.1038/nm.2160 20512126 PMC3107820

[B123] Musienko P. E. Bogacheva I. N. Savochin A. A. Kilimnik V. A. Gorskii O. V. Nikitin O. A. (2013). [Non-invasive transcutaneous spinal cord stimulation facilitates locomotor activity in decerebrated and spinal cats]. *Ross. Fiziol. Zh. Im. I M Sechenova* 99 917–927. 25470942

[B124] Nadeau S. Jacquemin G. Fournier C. Lamarre Y. Rossignol S. (2010). Spontaneous motor rhythms of the back and legs in a patient with a complete spinal cord transection. *Neurorehabil. Neural Repair* 24 377–383. 10.1177/1545968309349945 20019383

[B125] Nielsen J. B. Crone C. Hultborn H. (2007). The spinal pathophysiology of spasticity? From a basic science point of view. *Acta Physiol.* 189 171–180. 10.1111/j.1748-1716.2006.01652.x 17250567

[B126] Noga B. R. Johnson D. M. G. Riesgo M. I. Pinzon A. (2009). Locomotor-activated neurons of the cat. I. Serotonergic innervation and co-localization of 5-HT 7, 5-HT 2A, and 5-HT 1A receptors in the thoraco-lumbar spinal cord. *J. Neurophysiol.* 102 1560–1576. 10.1152/jn.91179.2008 19571190 PMC2746795

[B127] Noga B. R. Johnson D. M. G. Riesgo M. I. Pinzon A. (2011). Locomotor-activated neurons of the cat. II. Noradrenergic innervation and colocalization with NEα 1a or NEα 2b receptors in the thoraco-lumbar spinal cord. *J. Neurophysiol.* 105 1835–1849. 10.1152/jn.00342.2010 21307324 PMC3075296

[B128] Paroschy K. L. Shefchyk S. J. (2000). Non-linear membrane properties of sacral sphincter motoneurones in the decerebrate cat. *J. Physiol.* 523 741–753. 10.1111/j.1469-7793.2000.00741.x 10718752 PMC2269836

[B129] Pearson K. G. (2008). Role of sensory feedback in the control of stance duration in walking cats. *Brain Res. Rev.* 57 222–227. 10.1016/j.brainresrev.2007.06.014 17761295

[B130] Pearson K. G. Jiang W. Ramirez J. M. (1992). The use of naloxone to facilitate the generation of the locomotor rhythm in spinal cats. *J. Neurosci. Methods* 42 75–81. 10.1016/0165-0270(92)90137-3 1405735

[B131] Pearson K. G. Rossignol S. (1991). Fictive motor patterns in chronic spinal cats. *J. Neurophysiol.* 66 1874–1887. 10.1152/jn.1991.66.6.1874 1812222

[B132] Perreault M. C. Angel M. J. Guertin P. McCrea D. A. (1995). Effects of stimulation of hindlimb flexor group II afferents during fictive locomotion in the cat. *J. Physiol.* 487 211–220. 10.1113/jphysiol.1995.sp020872 7473250 PMC1156610

[B133] Perret C. Cabelguen J. Orsal D. (1988). “Analysis of the pattern of activity in “‘knee flexor”’ motoneurons during locomotion in the cat,” in *Stance and Motion: Facts and Concepts*, eds Gurfinkel V. S. Ioffe M. E. Massion J. Roll J. P. (Boston, MA: Springer), 133–141. 10.1007/978-1-4899-0821-6_12

[B134] Pierrot-Deseilligny E. Bergego C. Katz R. Morin C. (1981). Cutaneous depression of Ib reflex pathways to motoneurones in man. *Exp. Brain Res.* 42 351–361. 10.1007/BF00237500 7238675

[B135] Pozza M. E. Stella J. L. Chappuis-Gagnon A.-C. Wagner S. O. Buffington C. A. T. (2008). Pinch-induced behavioral inhibition (‘clipnosis’) in domestic cats. *J. Feline Med. Surg.* 10 82–87. 10.1016/j.jfms.2007.10.008 18222719 PMC10911153

[B136] Prévinaire J. G. Soler J. M. Perrigot M. (1998). Is there a place for pudendal nerve maximal electrical stimulation for the treatment of detrusor hyperreflexia in spinal cord injury patients? *Spinal Cord* 36 100–103. 10.1038/sj.sc.3100440 9494999

[B137] Prévinaire J. G. Soler J. M. Perrigot M. Boileau G. Delahaye H. Schumacker P. (1996). Short-term effect of pudendal nerve electrical stimulation on detrusor hyperreflexia in spinal cord injury patients: importance of current strength. *Spinal Cord* 34 95–99. 10.1038/sc.1996.17 8835034

[B138] Rossignol S. Dubuc R. Gossard J.-P. (2006). Dynamic sensorimotor interactions in locomotion. *Physiol. Rev.* 86 89–154. 10.1152/physrev.00028.2005 16371596

[B139] Rossignol S. Frigon A. (2011). Recovery of locomotion after spinal cord injury: some facts and mechanisms. *Annu. Rev. Neurosci.* 34 413–440. 10.1146/annurev-neuro-061010-113746 21469957

[B140] Sackman J. E. Sims M. H. (1990). Electromyographic evaluation of the external urethral sphincter during cystometry in male cats. *Am. J. Vet. Res.* 51 1237–1241. 2386321

[B141] Sasaki M. (1991). Membrane properties of external urethral and external anal sphincter motoneurones in the cat. *J. Physiol.* 440 345–366. 10.1113/jphysiol.1991.sp018712 1804967 PMC1180156

[B142] Schomburg E. D. (1990). Spinal sensorimotor systems and their supraspinal control. *Neurosci. Res.* 7 265–340. 10.1016/0168-0102(90)90008-3 2156196

[B143] Schomburg E. D. Petersen N. Barajon I. Hultborn H. (1998). Flexor reflex afferents reset the step cycle during fictive locomotion in the cat. *Exp. Brain Res.* 122 339–350. 10.1007/s002210050522 9808307

[B144] Schouenborg J. Holmberg H. Weng H.-R. (1992). Functional organization of the nociceptive withdrawal reflexes: II. Changes of excitability and receptive fields after spinalization in the rat. *Exp. Brain Res.* 90 469–478. 10.1007/BF00230929 1426107

[B145] Shefchyk S. J. (1989). The effects of lumbosacral deafferentation on pontine micturition centre-evoked voiding in the decerebrate cat. *Neurosci. Lett.* 99 175–180. 10.1016/0304-3940(89)90285-1 2748009

[B146] Shefchyk S. J. (2002). Spinal cord neural organization controlling the urinary bladder and striated sphincter. *Prog. Brain Res.* 137 71–82. 10.1016/S0079-6123(02)37008-0 12440360

[B147] Shefchyk S. J. (2006). Spinal mechanisms contributing to urethral striated sphincter control during continence and micturition: ‘How good things might go bad. *Prog. Brain Res.* 152 85–95. 10.1016/S0079-6123(05)52006-5 16198695

[B148] Shefchyk S. J. Espey M. J. Carr P. Nance D. Sawchuk M. Buss R. (1998). Evidence for a strychnine-sensitive mechanism and glycine receptors involved in the control of urethral sphincter activity during micturition in the cat. *Exp. Brain Res.* 119 297–306. 10.1007/s002210050345 9551830

[B149] Shik M. L. Severin F. V. Orlovskii G. N. (1966). [Control of walking and running by means of electric stimulation of the midbrain]. *Biofizika* 11 659–666. 6000625

[B150] Sie J. A. Blok B. F. de Weerd H. Holstege G. (2001). Ultrastructural evidence for direct projections from the pontine micturition center to glycine-immunoreactive neurons in the sacral dorsal gray commissure in the cat. *J. Comp. Neurol.* 429 631–637. 10.1002/1096-9861(20010122)429:4<631::aid-cne9>3.0.co;2-m 11135240

[B151] Smith J. C. Feldman J. L. Schmidt B. J. (1988). Neural mechanisms generating locomotion studied in mammalian brain stem-spinal cord *in vitro*. *FASEB J.* 2 2283–2288. 10.1096/fasebj.2.7.2450802 2450802

[B152] Stecina K. Quevedo J. McCrea D. A. (2005). Parallel reflex pathways from flexor muscle afferents evoking resetting and flexion enhancement during fictive locomotion and scratch in the cat: parallel reflexes from flexor muscle afferents. *J. Physiol.* 569 275–290. 10.1113/jphysiol.2005.095505 16141269 PMC1464219

[B153] Strauss I. Lev-Tov A. (2003). Neural pathways between sacrocaudal afferents and lumbar pattern generators in neonatal rats. *J. Neurophysiol.* 89 773–784. 10.1152/jn.00716.2002 12574455

[B154] Tai C. Miscik C. L. Ungerer T. D. Roppolo J. R. de Groat W. C. (2006). Suppression of bladder reflex activity in chronic spinal cord injured cats by activation of serotonin 5-HT1A receptors. *Exp. Neurol.* 199 427–437. 10.1016/j.expneurol.2006.01.007 16488413

[B155] Tai C. Shen B. Wang J. Chancellor M. B. Roppolo J. R. de Groat W. C. (2008). Inhibitory and excitatory perigenital-to-bladder spinal reflexes in the cat. *Am. J. Physiol. Renal Physiol.* 294 F591–F602. 10.1152/ajprenal.00443.2007 18160624 PMC3405732

[B156] Tax A. A. Van Wezel B. M. Dietz V. (1995). Bipedal reflex coordination to tactile stimulation of the sural nerve during human running. *J. Neurophysiol.* 73 1947–1964. 10.1152/jn.1995.73.5.1947 7623093

[B157] Thor K. B. Roppolo J. R. Degroat W. C. (1983). Naloxone induced micturition in unanesthetized paraplegic cats. *J. Urol.* 129 202–205. 10.1016/S0022-5347(17)51984-9 6681849

[B158] Van der Horst V. G. J. M. Holstege G. (1998). Sensory and motor components of reproductive behavior: pathways and plasticity. *Behav. Brain Res.* 92 157–167. 10.1016/S0166-4328(97)00188-5 9638958

[B159] Vanderhorst V. G. Holstege G. (1997). Organization of lumbosacral motoneuronal cell groups innervating hindlimb, pelvic floor, and axial muscles in the cat. *J. Comp. Neurol.* 382 46–76. 10.1002/(sici)1096-9861(19970526)382:1<46::aid-cne4>3.0.co;2-k 9136811

[B160] Viala G. Buser P. (1974). [Inhibition of spinal locomotor activity by a special method of somatic stimulation in rabbits]. *Exp. Brain Res.* 21 275–284. 10.1007/BF00235747 4442490

[B161] Viala G. Orsal D. Buser P. (1978). Cutaneous fiber groups involved in the inhibition of fictive locomotion in the rabbit. *Exp. Brain Res.* 33 257–267. 10.1007/BF00238064 700006

[B162] Wada N. Hamada K. Nishikawa H. (1996). Rhythmic discharges recorded from tail muscle nerves after injection of nialamide and L-DOPA solution in spinalized cats. *Arch. Ital. Biol.* 134 201–205. 8741228

[B163] Wada N. Sugita S. Kolblinger G. (1990). Spinal cord location of the motoneurons innervating the tail muscles of the cat. *J. Anat.* 173 101–107. 2074215 PMC1256085

[B164] Wheeler J. S. Walter J. S. Zaszczurynski P. J. (1992). Bladder inhibition by penile nerve stimulation in spinal cord injury patients. *J. Urol.* 147 100–103. 10.1016/S0022-5347(17)37145-8 1729491

[B165] Whelan P. Bonnot A. O’Donovan M. J. (2000). Properties of rhythmic activity generated by the isolated spinal cord of the neonatal mouse. *J. Neurophysiol.* 84 2821–2833. 10.1152/jn.2000.84.6.2821 11110812

[B166] Wolpaw J. R. (2018). The negotiated equilibrium model of spinal cord function: the spinal cord: a negotiated equilibrium. *J. Physiol.* 596 3469–3491. 10.1113/JP275532 29663410 PMC6092289

[B167] Yoo P. B. Klein S. M. Grafstein N. H. Horvath E. E. Amundsen C. L. Webster G. D. (2007). Pudendal nerve stimulation evokes reflex bladder contractions in persons with chronic spinal cord injury. *Neurourol. Urodyn.* 26 1020–1023. 10.1002/nau.20441 17480024

[B168] Zehr E. P. Hesketh K. L. Chua R. (2001). Differential regulation of cutaneous and H-reflexes during leg cycling in humans. *J. Neurophysiol.* 85 1178–1184. 10.1152/jn.2001.85.3.1178 11247987

[B169] Zehr E. P. Stein R. B. (1999). What functions do reflexes serve during human locomotion? *Prog. Neurobiol.* 58 185–205. 10.1016/S0301-0082(98)00081-1 10338359

